# The influence of L‐proline and fulvic acid on oxidative stress and semen quality of buffalo bull semen following cryopreservation

**DOI:** 10.1002/vms3.1158

**Published:** 2023-05-17

**Authors:** Negin Ramazani, Farid Mahd Gharebagh, Ali Soleimanzadeh, Halil Ozancan Arslan, Esin Keles, Desislava Georgieva Gradinarska‐Yanakieva, Damla Arslan‐Acaröz, Mahdi Zhandi, Alper Baran, Esmail Ayen, Dursun Ali Dinç

**Affiliations:** ^1^ Researcher in Biology and Animal Reproduction Urmia Iran; ^2^ Department of Theriogenology, Faculty of Veterinary Medicine Urmia University Urmia Iran; ^3^ Republic of Turkey Ministry of Agriculture and Foresty International Center for Livestock Research and Training Ankara Turkey; ^4^ Department of Reproductive Biotechnologies and Cryobiology of Gametes Institute of Biology and Immunology of Reproduction ‘Acad. Kiril Bratanov’ at Bulgarian Academy of Sciences Sofia Bulgaria; ^5^ Faculty of Veterinary Medicine Department of Biochemistry Afyon Kocatepe University Afyonkarahisar Turkey; ^6^ ACR Bio Food and Biochemistry Research and Development Afyonkarahisar Turkey; ^7^ Faculty of Veterinary Medicine Kyrgyz‐Turkish Manas University Bishkek Kyrgyzstan; ^8^ Faculty of Agriculture, Department of Animal Science College of Agriculture and Natural Resources University of Tehran Karaj Iran; ^9^ Faculty of Veterinary Medicine Department of Reproduction and Artificial Insemination Clinical Sciences, Istanbul University‐Cerrahpasa Avcilar‐Istanbul Turkey; ^10^ Faculty of Veterinary Medicine Department of Obstetrics and Gynecology University of Selcuk Konya Turkey

**Keywords:** Azari water buffalo, fulvic acid, L‐proline, post‐thawed semen

## Abstract

**Background:**

This study investigates the effects of cryopreservation and supplementation of Azeri water buffalo's semen with proline (Lp) and fulvic acid (FA).

**Objectives:**

Therefore, this study aimed to assess motility parameters, sperm viability, oxidative stress parameters, and DNA damage to detect the optimum concentrations of Lp and FA for buffalo semen cryopreservation.

**Methods:**

Thirty semen samples of three buffalo bulls were diluted in Tris‐egg yolk extender and divided into 12 equal groups including control (C), Lp‐10, Lp‐20, Lp‐40, Lp‐60, Lp‐80 (containing 10, 20, 40, 60, 80 mM L‐proline, respectively), FA‐0.2, FA‐0.5, FA‐0.8, FA‐1.1, FA‐1.4 and FA‐1.7 (containing 0.2%, 0.5%, 0.8%, 1.1%, 1.4% and 1.7% fulvic acid, respectively).

**Results:**

The velocity parameters, TM and PM were improved by FA‐1.7, FA‐1.4, Lp‐40 and Lp‐60 groups compared to the C group but no significant difference was found regarding the amplitude of lateral head displacement and straightness compared to the control groups. The percentage of sperm viability and PMF were increased by FA‐1.7, FA‐1.4, FA‐1.1, Lp‐40 and Lp‐60 groups compared to C group, while in terms of sperm DNA damage FA‐1.7, FA‐1.4, FA‐1.1, Lp‐10, Lp‐20, Lp‐40 and Lp‐60 groups showed better results compared to C group. The results also showed that FA‐1.7, FA‐1.4, FA‐1.1, Lp‐20, Lp‐40 and Lp‐60 groups could improve TAC, SOD, GSH and decrease MDA levels. Also, FA‐1.7, FA‐1.4, Lp‐20 and Lp‐40 groups could improve GPx levels but just FA‐1.7, and Lp‐40 groups could improve CAT levels compared to C group.

**Conclusions:**

Thus, it can be concluded that L‐proline and fulvic acid supplementations can improve the quality parameters of post‐thawed buffalo bull semen.

## Graphical abstract

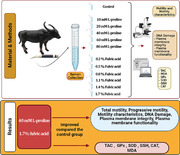

## 1 INTRODUCTION

Genetic diversity and characteristics of native breeds are recognised as valuable resources that cannot be recovered if extinct (Antkowiak et al., 2012). In addition to the physical protection of native species and ecotypes, the protection of their genetic pool should also be considered (Aminafshar et al., 2008). Azari ecotype is small in size with black skin and high‐quality milk production rate as well as high fat and a long milking period per year (Borghese & Mazzi, 2005; Pour Azary et al., 2004). This native endangered ecotype may further proceed to extinction in the future due to climate change. Therefore, it necessitates preserving genetic reserves of the buffalo that is considered as a vital genetic source in the production of milk and other products. Therefore, sperm freezing of this valuable ecotype is very important (Tavakolian, 2000).

Moreover, cryopreservation and thawing of semen produces reactive oxygen species (ROS), thus exposing the sperm to oxidative stress. The physiological levels of ROS affect the fertilisation potency and capacitation and acrosomal reaction of spermatozoa; at extremely high levels, ROS lead to apoptosis, decreased motility, chromatin damage, and impaired fertilisation ability (Elsayed et al., 2019). According to previous evidence (Izanloo et al., 2022; Partyka et al., 2011), it also increases sperm susceptibility to lipid peroxidation (LPO). Glutathione peroxidase (GPx), glutathione reductase, superoxide dismutase (SOD), and catalase (CAT) as enzymatic or natural antioxidants, as well as glutathione (GSH), ascorbic acid, carotenoids, vitamin E, selenium, coenzyme Q, and zinc as nonenzymatic or synthetic antioxidants have a defence mechanism against free radicals and LPO (Kasimanickam et al., 2006; Tuncer et al., 2010; Partyka et al., 2015; Papas et al., 2020, 2019).

Buffalo semen has an anti‐oxidative stress system that includes enzymatic and nonenzymatic antioxidants (Turaja et al., 2019), but during cryopreservation, due to the low concentration of these antioxidants in semen, the endogenous defence system is insufficient for countering this stress (Ansari et al., 2012). Low temperatures during the buffalo semen freezing and thawing cycle cause excessive adenosine triphosphate consumption, cell membrane lesions, DNA apoptosis, increased osmotic pressure, early acrosome reactions, and oxidative stress, reducing sperm motility and fertilisation capacity (De Lamirande et al., 1997; Tariq et al., 2015; Ezz et al., 2017). Therefore, an improvement in sperm cryopreservation leads to increased productivity in the breeding program of this animal species (Topraggaleh et al., 2014). According to some studies, antioxidants protect cells against oxidative damage and are also essential for sperm integrity and motility. For this reason, adding antioxidants to buffalo semen is necessary to improve fertilisation capacity and semen quality (Beheshti et al., 2011; Ansari et al., 2012; Saeed et al., 2016; Soleimanzadeh et al., 2020).

L‐proline is one of the essential amino acids that has proteinogenic properties (Moradi et al., 2022); it also has various functions such as being an osmoprotectant, stabilising cellular structures and enzymes, scavenging ROS and keeping up redox balance in adverse situations (Meena et al., 2019). Some researchers have reported that L‐proline has antioxidant and natural osmoprotective properties in relation to the destructive effects of freezing on various species, including human, donkey and ram sperm as well as mice oocytes (Sangeeta et al., 2015; Zhang et al., 2016; Li et al., 2021; Moradi et al., 2022).

Fulvic acids, a type of humic acids, are organic substances obtained from peat. Fulvic acids are often found in soil and natural water systems and have greater biological activities and are soluble in both acid and alkali solutions (Islam et al., 2005; Bai et al., 2013). On the other hand, Fulvic acids are rich in many reactive functional groups (phenols, quinones and hydroxyl), and these groups cause anti‐oxidative activity (Chang et al., 2013; Jayasooriya et al., 2016). It has been showed that humic and fulvic acid could improve sexual desire and semen quality under heat‐stress conditions (Abdel‐Khalek et al., 2019). On the other hand, Xiao and coworkers observed that fulvic acid have a cryoprotective effect on freezing goat buck semen (Xiao et al., 2018).

Today, although the technology of freezing and preserving semen for artificial insemination, especially in bulls and buffalo, has advanced beyond other species, there are still prominent gaps in the foundation of this knowledge and technology. Still, sperm survival rates after thawing are low and vary considerably among breeding bulls. These weaknesses are significant because they prevent progress in the fundamental science of reproductive biotechnology. However, the protective effects of L‐proline and fulvic acids on buffalo semen are still unknown. Hence, this study aims to determine the effects of L‐proline and fulvic acid supplementations to the extender following the freezing of Azeri water buffalo, on sperm characteristics and oxidative stress parameters.

## 2 MATERIAL AND METHODS

### 2.1 Chemicals

All required chemical compounds were obtained from Sigma (St. Louis, MO, USA) and Merck (Darmstadt, Germany).

### 2.2 Collection and processing of the semen

All animals were healthy and under the same management conditions. The artificial vagina method was used for semen collection twice a week. Overall, 30 ejaculations from three Iranian water buffalo bulls (*Bubalus bubalis*, Azari ecotype), the fertility of which had already been confirmed, were obtained during five weeks. The samples were examined for sperm quality; only those with a concentration greater than 500 × 10^6^ spermatozoa/mL, volume between 2–6 mL, total motility greater than 65% and abnormal morphology less than 20% per ejaculation were considered normal and investigated.

A standard Tris‐based extender (Tris: 2.66 g per 100 mL, citric acid: 1.47 g per 100 mL, glucose: 0.63 g per 100 mL, egg yolk: 20% (v/v), glycerol: 7% v/v, penicillin: 1 mg per 100 mL, streptomycin: 1 mg per 100 mL and pH: 6.8), according to previous research (Topraggaleh et al., 2014), was used as base extender in this study. In each replicate, semen samples (*n* = 3) were pooled and equally divided to 12 parts and each part was diluted to a final concentration of 15 × 10^6^ spermatozoa/mL (Tahmasbian et al., 2022) with one of the following extenders including: Group 1: Control (Tris‐based extender without antioxidant); Lp‐10: Tris‐based extender + L‐proline (10 mM); Lp‐20: Tris‐based extender + L‐proline (20 mM); Lp‐40: Tris‐based extender + L‐proline (40 mM); Lp‐60: Tris‐based extender + L‐proline (60 mM); Lp‐80: Tris‐based extender + L‐proline (80 mM) (Li et al., 2021); FA‐0.2: Tris‐based extender + fulvic acid (0.2% w/w); FA‐0.5: Tris‐based extender + fulvic acid (0.5% w/w); FA‐0.8: Tris‐based extender + fulvic acid (0.8% w/w); FA‐1.1: Tris‐based extender + fulvic acid (1.1% w/w); FA‐1.4: Tris‐based extender + fulvic acid (1.4% w/w); FA‐1.7: Tris‐based extender + fulvic acid (1.7% w/w) (Xiao et al., 2018). The straws were put in a refrigerator set at 4˚C for 2 h and then semen was frozen according to the following protocol: from + 4°C to −12˚C at a rate of – 4˚C /min, from – 12˚C to – 40˚C at a rate of – 40˚C/min and from – 40˚C up to – 140˚C at a rate of – 50 ˚C/min. Then they were transferred into goblets floating in liquid nitrogen. After thawing at 38˚C for 30 s, three samples from each frozen pie were analysed for the quality parameters of semen (Soleimanzadeh et al., 2020).

### 2.3 Semen analysis

#### 2.3.1 Motility and motion parameters

The motility characteristics of semen were evaluated by a CASA system (Test Sperm 3.2; Videotest, St. Petersburg, Russia), using 10 microliters of the thawed semen. Various parameters such as total motility (TM, %), progressive motility (PM, %), curvilinear velocity (VCL, µm/s), straight‐line velocity (VSL, µm/s), average path velocity (VAP, µm/s), straightness (STR, %), linearity (LIN, %), amplitude of lateral head displacement (ALH, µm/s) and beat‐cross frequency (BCF, Hz) were examined by studying at least 500 spermatozoa in five microscopic fields (Table 1).

**TABLE 1:** Parameter settings for the CASA.

Parameter	Setting
Frame rate	60 Hz
Duration of capture	1 s
Stage temperature rate	37°C
Minimum cell size	5 pixels
Cell size	5 pixels
Minimum contrast	80
Cell intensity	70 pixels
Chamber type	Slide coverslip (22 × 22 mm)
Volume per slide	7 µL
Chamber depth	≈ 20 µm
Minimum number of field analysis	500 cells
Sample dilution	20 × 10^6^
Image type	Phased contrast

#### 2.3.2 DNA damage evaluation

DNA fragmentation is one of the damages to sperm DNA and causes infertility. Acridine orange staining was performed to investigate DNA damage and identify denatured, double‐stranded DNA segments in the chromatin of the sperm at a low pH rate (Narayana et al., 2002). A thick smear was fixed for 2 h in a Carnoy's fixative consisting of methanol and acetic acid (1: 3). Then, it was extracted and dried at room temperature for 5 min. Next, it was placed in the stock solution containing 1 mg of orange acridine and 1000 mL of distilled water, and the resulting mixture was placed in a dark place at 4^°^C for 5 min. A fluorescent microscope (Model GS7, Nikon Co., Tokyo, Japan) in ×200 magnification was used to evaluate sperms at 490 nm. Among them, yellow and red sperms were considered damaged and abnormal (Talebi et al., 2011).

#### 2.3.3 Sperm plasma membrane functionality

Hypoosmotic swelling test (HOST) was applied to identify spermatozoa with intact membranes. For this purpose, 10 µL of semen was diluted in a 100 µL of the hypoosmotic solution )fructose 1.35 g and sodium citrate 0.73 g) solution and the resulting mixture was incubated at 37°C (M. Khan & Ijaz, 2007). Subsequently, a contrast‐phase microscope (Olympus, BX41, Tokyo, Japan) with 200× magnification was employed to examine the PM of the sperm. In general, 200 curled or straight sperms were counted.

#### 2.3.4 Sperm viability

Sperm viability was evaluated based on a WHO protocol by eosin‐nigrosine staining (WHO, 1999). Eosin (Merck, Darmstadt, Germany) and Nigrosine (Merck, Darmstadt, Germany) were prepared in distilled water. This was done by mixing two volumes of 1% eosin with one volume of sperm, and they were counted with a light microscope in ×200 magnification (Model CHT, Olympus optical Co. Ltd). The red stained spermatozoa were considered nonviable, while the viable ones were colourless.

### 2.4 Assessment of enzymatic antioxidants activity

Semen biochemical analyses were performed using six straws (one for each extender). After thawing, 120 µL of the semen was centrifuged at 1600 × *g* at 25°C for 5 min. The supernatant was removed, and 360 µL of 1% Triton X‐100 was precipitated for 20 min for extraction. The resulting mixture was centrifuged at 4000 × *g* at 25°C for 30 min. The suspending of the precipitate was repeated once more, and the supernatant was obtained as the crude extract of the enzymes present in the sperm.

#### 2.4.1 Malondialdehyde (MDA)

An MDA test kit (Nalondi™ Lipid Peroxidation Assay Kit (NID), Navand Salamat Company, Urmia, Iran) was applied to evaluate oxidative stress by assessing MDA concentration. A spectrophotometer (Thermo Fisher Scientific; Waltham, MA, USA) and a standard curve were used to read the supernatant at 523 nm and to express the amount of MDA as nmol/mL, respectively.

#### 2.4.2 Total antioxidant capacity (TAC)

A TAC kit was employed to measure TAC (Naxifer™ – Cat# NS‐15012; Total Antioxidant Capacity Assay Kit‐TAC, Navand Salamat Company, Urmia, Iran). The values of TAC were expressed as mmol/L.

#### 2.4.3 Glutathione peroxidase (GPx)

A GPx kit (Nagpix™ – CAT# NS‐15083, Glutathione Peroxidase Activity Assay Kit; Navand Salamat Company, Urmia, Iran (was utilised to estimate GPx in semen after thawing according to the manufacturer's instructions. GPx was expressed as mU/mL.

#### 2.4.4 Superoxide dismutase (SOD)

A SOD assay kit (Nasdox ™ Superoxide Dismutase Assay Kit; Navand Salamat Company, Urmia, Iran) was applied to evaluate SOD. The reduction of colour development at 405 nm was used to determine the SOD activity, which is considered an inhibitory activity. Plasma SOD activity was expressed as U/mL.

#### 2.4.5 Catalase evaluation

A 2‐step method using a commercial CAT kit (Nactaz™ Catalase Activity Assay Kit; Navand Salamat Company, Urmia, Iran) was considered to evaluate the activity of CAT. An absorbance rate of 550 nm was taken into consideration for determining CAT after incubation for 10 min at room temperature. The CAT activity in the plasma was expressed as U/mL.

#### 2.4.6 Glutathione evaluation

A GSH commercial kit (NarGul™ – Glutathione Assay Kit‐GSH; Navand Salamat Company, Urmia, Iran) was utilised for GSH activity, and its value was recorded as U/L.

### 2.5 Statistical analysis

The obtained data were analysed by SPSS (Version 26.0; IBM CO., Chicago, USA) using one‐way ANOVA and Tukey's post hoc test and *p ≤* 0.05 was considered statistically significant.

## 3 RESULTS

According to the results obtained by CASA (Table 2), adding FA‐1.7, FA‐1.4, FA‐1.1, Lp‐20 and Lp‐40 could improve TM and PM compared to C group (*p* ≤ 0.05; Table 2). The lowest percentage of TM was observed in Lp‐80 and FA‐0.2 (*p* ≤ 0.05; Table 2).

**TABLE 2:** Effects of different concentrations of L‐proline (Lp) and fulvic acid (FA) on buffalo sperm total and progressive at post‐thawed stage of cryopreservation. Values are expressed as mean ± SEM.

Parameters	Control	Lp‐10	Lp‐20	Lp‐40	Lp‐60	Lp‐80	FA‐0.2	FA‐0.5	FA‐0.8	FA‐1.1	FA‐1.4	FA‐1.7
Total motility (%)	55.47±1.19^de^	57.41±1.16^d^	64.59±1.76^bc^	68.45±1.69^ab^	62.30±1.54^c^	51.18±1.25^f^	48.11±1.37^g^	53.72±1.48^ef^	58.24±1.56^d^	64.07±1.89^bc^	66.21±1.15^b^	71.49±1.31^a^
Progressive motility (%)	25.31±1.84^d^	25.74±1.52^d^	31.26±1.38^c^	36.43±1.75^b^	30.14±1.96^c^	19.02±1.47^e^	15.69±1.20^f^	22.10±1.33^d^	26.77±1.09^d^	30.07±1.50^c^	33.84±1.16^bc^	40.23±1.47^a^

Control (C): Tris‐based extender without antioxidant; Lp‐10: Tris‐based extender + L‐proline (10 mM); Lp‐20: Tris‐based extender + L‐proline (20 mM); Lp‐40: Tris‐based extender + L‐proline (40 mM); Lp‐60: Tris‐based extender + L‐proline (60 mM); Lp‐80: Tris‐based extender + L‐proline (80 mM); FA‐0.2: Tris‐based extender + fulvic acid (0.2%); FA‐0.5: Tris‐based extender + fulvic acid (0.5%); FA‐0.8: Tris‐based extender + fulvic acid (0.8%); FA‐1.1: Tris‐based extender + fulvic acid (1.1%); FA‐1.4: Tris‐based extender + fulvic acid (1.4%); FA‐1.7: Tris‐based extender + fulvic acid (1.7%). Different superscripts within the same row demonstrate significant differences (*p* ≤ 0.05).

Results presented in Table 3 showed motility characteristics (VCL, VSL, VAP, STR, LIN and BCF) in post‐thawed buffalo semen. The values of VCL, BCF and LIN in FA‐1.7, FA‐1.4, FA‐1.1, Lp‐60, Lp‐40 and Lp‐20 groups showed an improvement compared to C group (*p* ≤ 0.05; Table 3), while Lp‐10 and FA‐0.8 groups showed no significant compared to C group (*p* > 0.05; Table 3). Also, the values of VCL and LIN in FA‐0.2 and Lp‐80 groups and BCF in FA‐0.5, FA‐0.2 and Lp‐80 groups showed decrease compared to C group (*p* ≤ 0.05; Table 3). The values of VAP in FA‐1.7, FA‐1.4, Lp‐40 and Lp‐20 groups and VSL in FA‐1.7, FA‐1.4, Lp‐60 and Lp‐40 groups showed an increase compared to C group (*p* ≤ 0.05; Table 3). According to the results, the values of ALH and STR showed no significant improvement in all treated groups compared to C group (*p* > 0.05; Table 3).

**TABLE 3:** Mean (±SEM) sperm velocity characteristics of frozen‐thawed buffalo semen after addition of different concentrations of L‐proline (Lp) and fulvic acid (FA) in semen extender.

Parameters	Control	Lp‐10	Lp‐20	Lp‐40	Lp‐60	Lp‐80	FA‐0.2	FA‐0.5	FA‐0.8	FA‐1.1	FA‐1.4	FA‐1.7
VAP (µm/s)	25.69±1.30^de^	25.20±1.65^de^	29.19±1.65^bc^	32.28±1.54^b^	28.57±1.31^bcd^	22.02±1.12^e^	22.35±1.47^e^	23.78±1.34^e^	26.17±1.79^cd^	27.33±1.22^cd^	31.96±1.39^b^	36.43±1.40^a^
VCL (µm/s)	39.41±1.65^e^	41.05±1.01^e^	46.20±1.12^cd^	51.65±1.73^b^	44.25±1.57^d^	35.38±0.95^f^	32.77±0.30^g^	40.11±1.28^e^	40.94±1.64^e^	42.56±1.10^d^	49.94±1.83^bc^	55.69±1.19^a^
VSL (µm/s)	20.93±1.03^cd^	21.57±1.64^bcd^	25.35±1.49^bc^	27.08±1.10^ab^	23.57±1.42^bc^	16.50±1.18^d^	17.35±1.61^d^	19.89±1.84^d^	21.33±1.17^cd^	22.83±1.25^c^	26.49±1.01^b^	30.17±1.33^a^
LIN (%)	43.20±1.75^e^	44.79±1.62^e^	51.72±1.56^cd^	55.72±1.69^b^	49.03±1.20^cd^	39.17±1.58^f^	37.93±1.05^f^	41.65±1.19^ef^	45.78±1.85^de^	48.27±1.64^d^	53.74±1.22^bc^	59.20±1.94^a^
ALH (µm/s)	2.45±0.11^a^	2.31±0.14^a^	2.46±0.18^a^	2.34±0.07^a^	2.23±0.18^a^	2.32±0.15^a^	2.25±0.73^a^	2.24±0.46^a^	2.33±0.70^a^	2.37±0.25^a^	2.30±0.19^a^	2.34±0.72^a^
STR (%)	72.73±2.28^a^	72.99±2.55^a^	72.53±1.41^a^	73.45±1.41^a^	72.67±1.22^a^	72.24±1.66^a^	72.68±1.50^a^	72.30±1.85^a^	72.55±1.57^a^	72.48±1.82^a^	72.64±1.15^a^	73.18±1.09^a^
BCF (Hz)	3.28±0.13^e^	3.24±0.17^e^	5.06±0.19^d^	7.21±0.16^b^	5.11±0.28^d^	2.54±0.12^f^	2.20±0.15^f^	2.77±0.13^f^	3.30±0.19^e^	5.33±0.25^d^	6.51±0.20^c^	8.09±0.23^a^

Control (C): Tris‐based extender without antioxidant; Lp‐10: Tris‐based extender + L‐proline (10 mM); Lp‐20: Tris‐based extender + L‐proline (20 mM); Lp‐40: Tris‐based extender + L‐proline (40 mM); Lp‐60: Tris‐based extender + L‐proline (60 mM); Lp‐80: Tris‐based extender + L‐proline (80 mM); FA‐0.2: Tris‐based extender + fulvic acid (0.2%); FA‐0.5: Tris‐based extender + fulvic acid (0.5%); FA‐0.8: Tris‐based extender + fulvic acid (0.8%); FA‐1.1: Tris‐based extender + fulvic acid (1.1%); FA‐1.4: Tris‐based extender + fulvic acid (1.4%); FA‐1.7: Tris‐based extender + fulvic acid (1.7%). Different superscripts within the same row demonstrate significant differences (*p* ≤ 0.05). VAP: average path velocity; VCL: curvilinear velocity; VSL: straight‐line velocity; LIN: linearity; ALH: amplitude of lateral Head displacement; BCF: beat‐cross frequency; STR: straightness.

**FIGURE 1:**
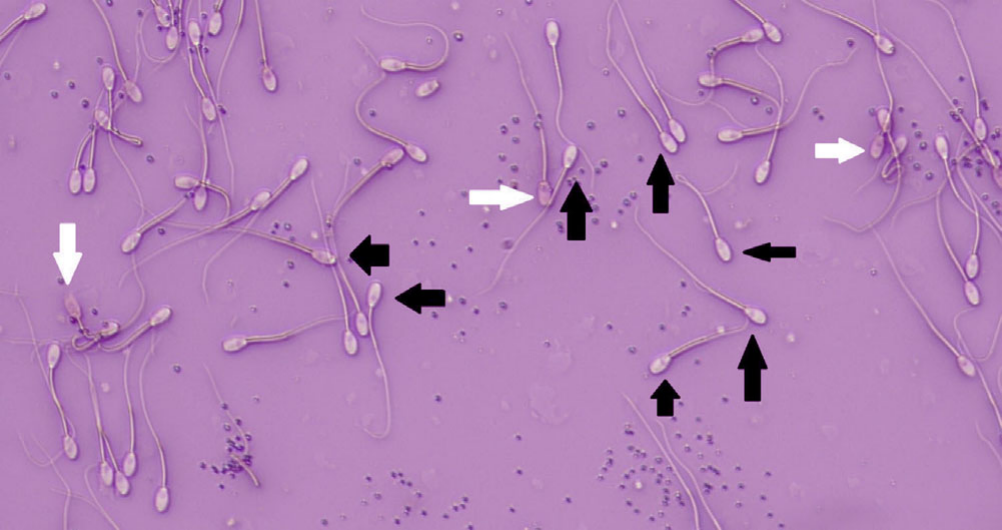
Sperm viability. White arrow – viable spermatozoa (colourless), black arrow – dead spermatozoa (red) (eosin/nigrosin, 200×).

**FIGURE 2:**
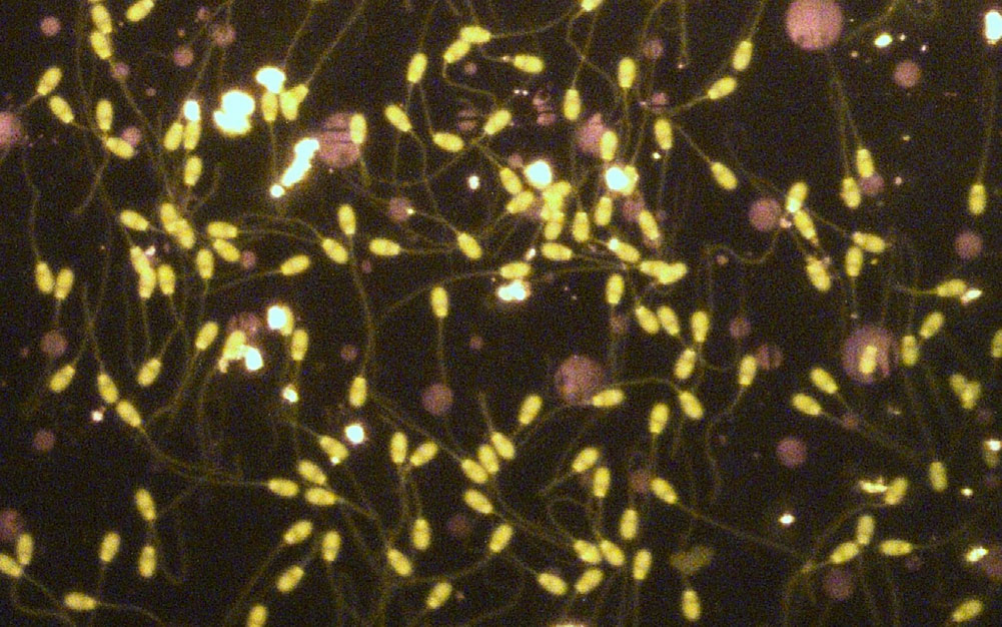
Buffalo spermatozoa DNA damage; normal spermatozoa (green) (acridine orange, 200×).

**FIGURE 3:**
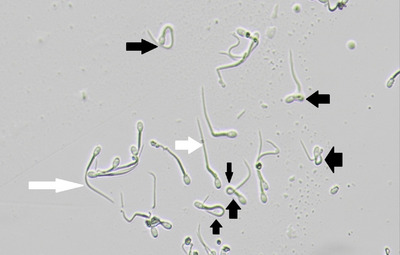
PM functionality. Black arrow – nonfunctional PM (spermatozoa with straight tails); white arrow – functional PM (spermatozoa with coiled tails) (200×).

The results showed that sperm viability in FA‐1.7, FA‐1.4, FA‐1.1, Lp‐60, Lp‐40 and Lp‐20 groups and PMF in FA‐1.7, FA‐1.4, FA‐1.1, Lp‐60 and Lp‐40 groups have been improved (*p* ≤ 0.05; Table 4; Figures 1 and 2). The results showed a decrease in DNA damage in FA‐1.7, FA‐1.4, FA‐1.1, FA‐0.8, Lp‐60, Lp‐40, Lp‐20 and Lp‐10 groups compared to C group (*p* ≤ 0.05; Table 4; Figure 3).

**TABLE 4:** Effect of different concentrations of L‐proline (Lp) and fulvic acid (FA) supplementation to DNA damage, sperm viability and plasma membrane functionality (PMF) (mean ± SEM) in frozen‐thawed buffalo semen.

Groups	Control	Lp‐10	Lp‐20	Lp‐40	Lp‐60	Lp‐80	FA‐0.2	FA‐0.5	FA‐0.8	FA‐1.1	FA‐1.4	FA‐1.7
Sperm viability (%)	66.83±2.15^de^	67.90±2.53^de^	74.58±2.46^bc^	76.19±2.63^b^	72.95±2.40^c^	60.68±2.05^f^	57.11±2.37^f^	64.70±2.95^e^	68.23±2.11^d^	73.49±2.65^bc^	74.10±2.03^bc^	80.79±2.42^a^
DNA damage (%)	9.30±0.18^c^	8.73±0.31^d^	6.65±0.70^e^	3.27±0.94^g^	6.09±0.27^e^	11.35±0.41^b^	12.47±0.21^a^	10.84±0.27^b^	8.10±0.19^d^	5.84±0.21^f^	3.31±0.25^g^	3.05±0.11^g^
PMF (%)	61.47±2.31^c^	62.67±2.29^c^	65.38±2.73^bc^	70.42±2.67^a^	66.74±2.12^b^	56.83±1.95^d^	55.74±1.43^d^	59.02±1.69^c^	63.79±2.15^bc^	66.31±2.37^b^	66.50±2.45^b^	71.48±2.19^a^

Control (C): Tris‐based extender without antioxidant; Lp‐10: Tris‐based extender + L‐proline (10 mM); Lp‐20: Tris‐based extender + L‐proline (20 mM); Lp‐40: Tris‐based extender + L‐proline (40 mM); Lp‐60: Tris‐based extender + L‐proline (60 mM); Lp‐80: Tris‐based extender + L‐proline (80 mM); FA‐0.2: Tris‐based extender + fulvic acid (0.2%); FA‐0.5: Tris‐based extender + fulvic acid (0.5%); FA‐0.8: Tris‐based extender + fulvic acid (0.8%); FA‐1.1: Tris‐based extender + fulvic acid (1.1%); FA‐1.4: Tris‐based extender + fulvic acid (1.4%); FA‐1.7: Tris‐based extender + fulvic acid (1.7%). Different superscripts within the same row demonstrate significant differences (*p* ≤ 0.05).

The TAC capacity was higher in the FA‐1.7, FA‐1.4, FA‐1.1, Lp‐60, Lp‐40 and Lp‐20 compared to C group (*p* ≤ 0.05; Figure 4A). According to the results, GPx activities inFA‐1.7, FA‐1.4, Lp‐40 and Lp‐20 improved compared to C group (*p* ≤ 0.05; Figure 4B). The values of SOD and GSH improved in FA‐1.7, FA‐1.4, FA‐1.1, Lp‐60, Lp‐40 and Lp‐20 groups compared to C group (*p* ≤ 0.05; Figure 4C and D). The values of CAT were higher in FA‐1.7 and Lp‐40 compared to C group (*p* ≤ 0.05). The values of MDA in FA‐1.7, FA‐1.4, FA‐1.1, Lp‐60, Lp‐40 and Lp‐20 were lower than C groups (*p* ≤ 0.05; Figure 4F).

FIGURE 4(a) Total antioxidant capacity (TAC); (b) glutathione peroxidase (GPx); (c) superoxide dismutase (SOD); (d) glutathione (GSH) activities; (e) catalase (CAT); (f) lipid peroxidation (MDA) of frozen‐thawed buffalo semen after supplementation of different concentrations of L‐proline (Lp) and fulvic acid (FA) to the semen extender. Control (c): Tris‐based extender without antioxidant; Lp‐10: Tris‐based extender + L‐proline (10 mM); Lp‐20: Tris‐based extender + L‐proline (20 mM); Lp‐40: Tris‐based extender + L‐proline (40 mM); Lp‐60: Tris‐based extender + L‐proline (60 mM); Lp‐80: Tris‐based extender + L‐proline (80 mM); FA‐0.2: Tris‐based extender + fulvic acid (0.2%); FA‐0.5: Tris‐based extender + fulvic acid (0.5%); FA‐0.8: Tris‐based extender + fulvic acid (0.8%); FA‐1.1: Tris‐based extender + fulvic acid (1.1%); FA‐1.4: Tris‐based extender + fulvic acid (1.4%); FA‐1.7: Tris‐based extender + fulvic acid (1.7%). Different superscripts within the same row demonstrate significant differences (*p* ≤ 0.05; mean ± SEM).

**Figure:**
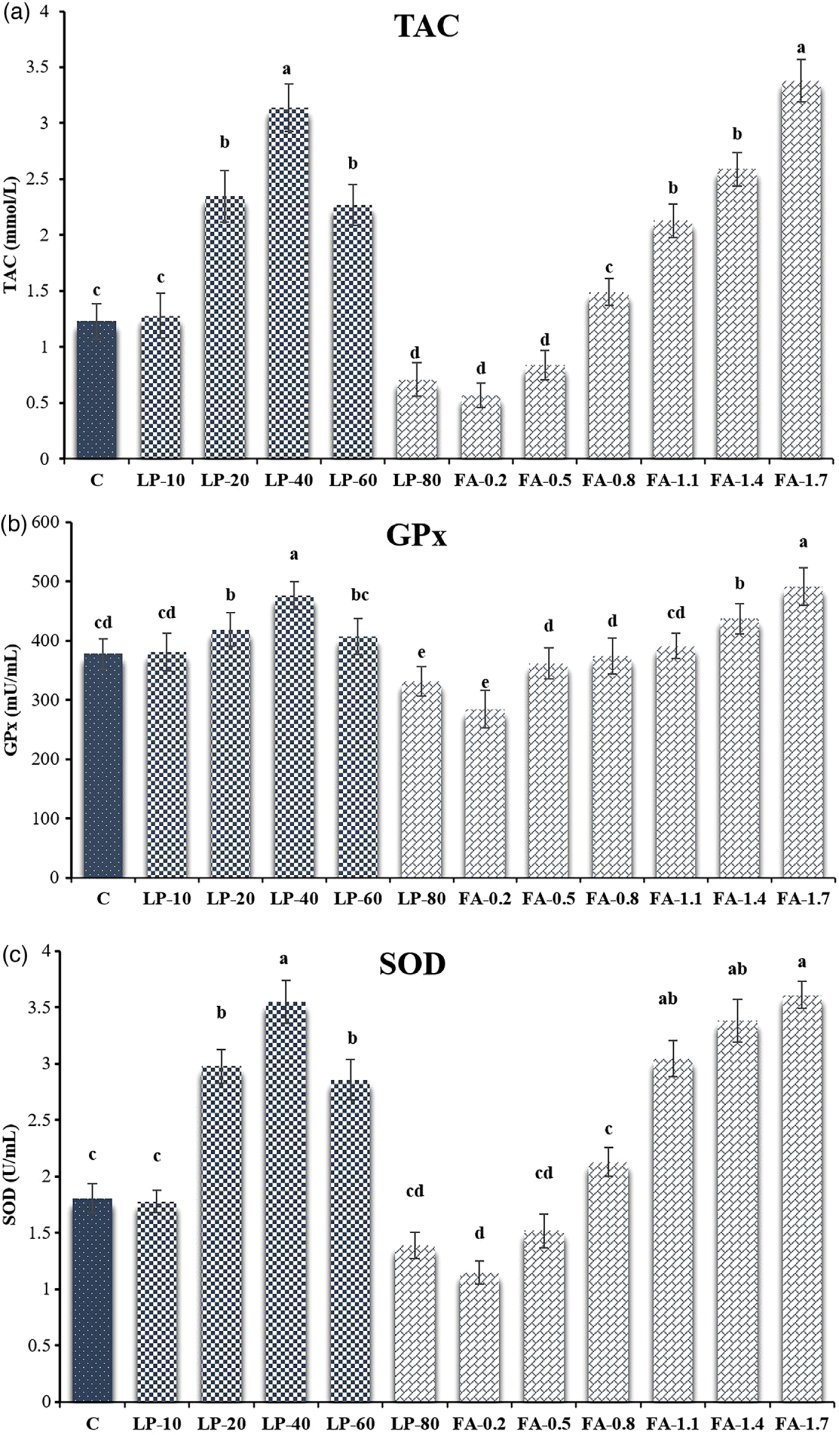

**Figure:**
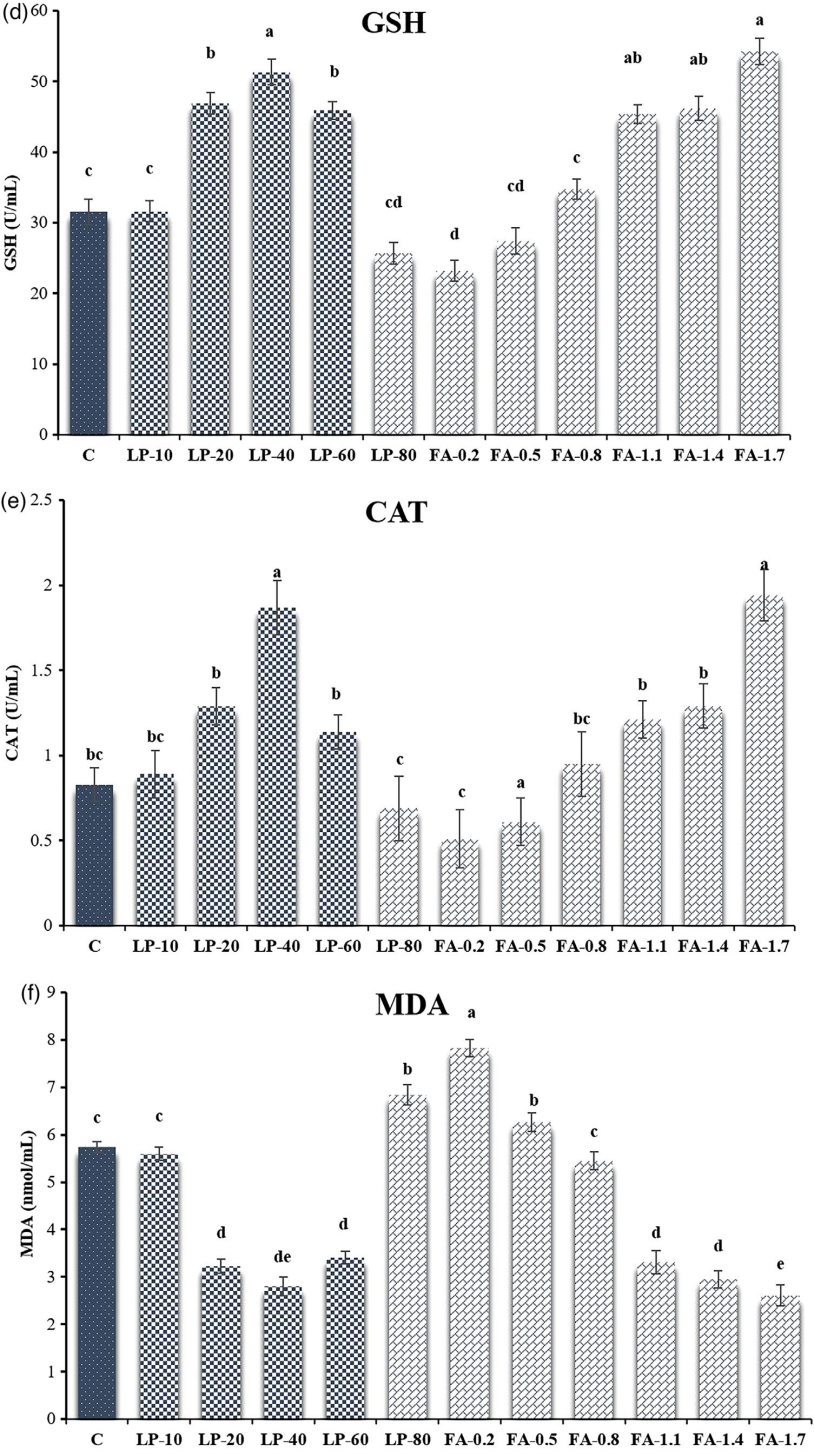

## 4 DISCUSSION

The present study demonstrated that using Lp and FA supplementation could improve some sperm quality parameters (including sperm motility and velocity parameters, sperm viability and PMF, DNA integrity and antioxidant profile). Similar to our study, it was demonstrated that using various antioxidants also improved post‐thawed semen quality parameters (Longobardi et al., 2017; Lone et al., 2018; Ahmed et al., 2020b; Soleimanzadeh et al., 2020).

The cryopreservation process leads to the overproduction of ROS, and to cope with this issue, and maintain the balance of ROS, antioxidants are necessary. Some studies showed that the fertility rate of cryopreserved semen is lower than that of the fresh sperm due to reduced nucleus and mitochondrial membrane integrity, plasma loss, protein denaturation, DNA damage, and oxidative stress (Ejaz et al., 2014; Ahmed et al., 2016). Reactive oxygen species are produced during the cryopreservation process, which reduces motility, viability, intracellular enzyme activity and sperm fertility potential (Selvaraju et al., 2008). In addition, the antioxidant system of the buffalo semen is insufficient for protecting the sperm from the harmful effects of oxidative stress during the cryopreservation process (Bansal & Bilaspuri, 2011). The production of free radicals and ROS is directly related to protein dephosphorylation. Therefore, it is assumed that the compounds used in this study maybe inhibiting protein dephosphorylation through the cAMP‐PKA pathway in semen during storage (Fu et al., 2018). Different enzymes such as CAT, GPx and reductase, and SOD are the main defenders of the sperm plasma membrane against ROS and LPO (Selvaraju et al., 2008). Xiao et al. (2018) showed that FA addition to semen extender improved antioxidant profile of post‐thawed goat sperm. In the current study, FA‐1.7, FA‐1.4, FA‐1.1, Lp‐60, Lp‐40 and Lp‐20 groups could improve TAC levels, SOD and GSH activities. Also, FA‐1.7, FA‐1.4, Lp‐40 and Lp‐20 could improve GPx activities and FA‐1.7 and Lp‐40 could improve CAT activities. While FA‐1.7, FA‐1.4, FA‐1.1, Lp‐60, Lp‐40 and Lp‐20 could decrease MDA levels compared to the C group. Therefore, it is assumed that the Lp and FA used in this study maybe inhibiting protein dephosphorylation during cryopreservation (Fu et al., 2018). In another study on buffalo spermatozoa, Iqbal et al. (2016) found that the activity of these enzymes was significantly improved after thawing by adding 30 mM trehalose to the extender. Based on the reports of Soleimanzadeh et al. (2020) the caffeic acid (100 µM) supplemented extender improved the antioxidant profile of buffalo semen after thawing. Reddy et al. (2010) also demonstrated that adding 50 mM taurine to the semen extender could significantly improve the antioxidant status.

Sperm motility and velocity parameters have positive correlation with sperm fertility and pregnancy in buffalo and cattle (Kasai et al., 2002; Kefer et al., 2009; Soleimanzadeh et al., 2020). Also, Shahzad et al. (2016) suggested that in order to check the fertility rate in buffalo, sperm motility rate can be evaluated after thawing, and the reason for that can be the damage to the sperm membrane that occurs during the cryopreservation process. The results of the current study showed that the addition of Lp and FA to extender could improve TM, PM and velocity parameters, except for ALH and STR, especially at FA‐1.7, FA‐1.4 and Lp‐40, which had the highest yield. Similarly, Sangeeta et al. (2015) reported that Lp at a concentration of 25 to 50 mM improved TM and PM in ram semen. Additionally, other study showed that the proline can increased sperm motility after thawing in buck semen (Farshad & Hosseini, 2013). Also, Li et al. (2021) observed that adding 30 mM of Lp to semen extender had favourable results on the TM of post‐thawed donkey sperm. In addition, our results are in agreement with Xiao et al. (2018) and Abdel‐Khalek et al. (2019) in bucks, who reported a beneficial effect of FA on sperm motility. These results can be explained by the fact that these compounds may protect the sperm membrane against protein dephosphorylation and cause them to improve sperm motility after freeze‐thawing process (Fu et al., 2018).

The main cause of sperm cell damage during freezing is the impairment in plasma membrane (Ugur et al., 2019). Sperm cryo‐injury occurs during cryopreservation due to changes in ice crystal damage. These injuries of sperm lead to decreased sperm viability and functionality (Khan et al., 2021). Also, in the course of the cooling process, limitations of phospholipid lateral motion induce a liquid‐to‐gel phase shift, making the plasma membrane rigid and fragile. These changes lead to lipid phase separation, and thus, the proteins are irreversibly clustered (De Leeuw et al., 1990). El‐Sheshtawy et al. (2008) found that antioxidants supplements can increase buffalo sperm viability after thawing. In a previous study, it has been reported that FA can improve sperm viability of goat spermatozoa during preservation (Xiao et al., 2018). Similar above‐mentioned studies, our study showed that FA‐1.7, FA‐1.4, FA‐1.1, Lp‐60 and Lp‐40 groups could be improved sperm viability and PMF in buffalo semen after cryopreservation. Other studies demonstrated that sperm viability could be improved by cysteine and glutamine (Topraggaleh et al., 2014), Royal jelly (Shahzad et al., 2016) or green tea extract (Ahmed et al., 2020a).

According to previous evidence, infertile males have more DNA damage than fertile males, and this damage may have a negative effect on male fertility (Narayana et al., 2002; Talebi et al., 2011). Sperm freezing, as an adverse process, has been shown to destabilise chromatin and lead to sperm DNA fragmentation in boars and birds (Fraser & Strzeżek, 2007; Gliozzi et al., 2011). Double‐stranded DNA breaks are due to high levels of ROS production, disruption of DNA repair enzymes and mechanical stress of DNA molecular genomic regions where chromatin density increases due to cell contraction (Bogle et al., 2017). Some studies have shown that DNA damage can be a cause of infertility in males (Narayana et al., 2002; Talebi et al., 2011). The results of current study showed that FA‐1.7, FA‐1.4, FA‐1.1, FA‐0.8, Lp‐60, Lp‐40, Lp‐20 and Lp‐10 groups could reduce DNA damage in buffalo semen after cryopreservation. In the present study, the concentration of 40 mM Lp had the greatest effect in minimising the amount of DNA damage. The high level of sperm DNA damage at higher doses (80 mM) of LP may be due to the fact that increasing the amount of L‐proline creates a toxic state for sperm. This is confirmed by the MDA values shown in this study, which increase the amount of malondialdehyde in the cells as the dose of L‐proline increases. In this respect, Topraggaleh et al. (2014) found that the concentration of 7.5 mM cysteine supplementation in the extender reduced amount of DNA damage in buffalo semen. In the study by Ejaz et al. (2014) adding 20 ng/mL arachidic acid to the extender also improved sperm chromatin integrity in buffalo semen. Dorostkar et al. (2012) reported that the addition of 2 mg/mL sodium selenite to extender reduced damage to buffalo sperm DNA, which is in line with our findings.

## 5 CONCLUSION

In conclusion, the addition of Lp and FA to semen extender could improve sperm motility and had antioxidant properties against ROS and free radicals (especially at the Lp‐40 and FA‐1.7 groups). It also improved sperm antioxidant capacity, reduced damage to sperm viability, and improved sperm PMF. Therefore, the addition of Lp and FA can improve the quality of frozen‐thawed semen in buffaloes. Further studies are warranted to determine the effects of Lp and FA supplementations into the Tris‐based extender on fertility.

## AUTHOR CONTRIBUTIONS

Negin Ramazani: data curation; methodology; software; supervision; writing – original draft; writing – review & editing. Farid Mahd Gharebagh: conceptualisation; data curation; methodology; software; writing – original draft; writing – review & editing. Ali Soleimanzadeh: conceptualisation; data curation; formal analysis; methodology; project administration; resources; software; writing – original draft; writing – review & editing. Esin Keles: methodology; writing – original draft; writing – review & editing. Desislava Georgieva Gradinarska‐Yanakieva: writing – original draft; writing – review & editing. Mahdi Zhandi: resources; software; writing – original draft; writing – review & editing. Alper Baran: methodology; resources; writing – original draft; writing – review & editing. Esmail Ayen: methodology; resources; supervision; writing – original draft; writing – review & editing. Dursun Ali Dinç: methodology; software; supervision; writing – original draft; writing – review & editing.

## CONFLICT OF INTEREST STATEMENT

None of the authors have any conflict of interest to declare.

## FUNDING

This research has not been financially supported.

## Data Availability

The data that support the findings of this study are available on request from the corresponding author. The data are not publicly available due to privacy or ethical restrictions.
